# Characterization of Rabbit Nucleotide-Binding Oligomerization Domain 1 (NOD1) and the Role of NOD1 Signaling Pathway during Bacterial Infection

**DOI:** 10.3389/fimmu.2017.01278

**Published:** 2017-10-10

**Authors:** Mengjiao Guo, Fahao Wu, Zhongfang Zhang, Guangen Hao, Rong Li, Ning Li, Yingli Shang, Liangmeng Wei, Tongjie Chai

**Affiliations:** ^1^College of Animal Science and Veterinary Medicine, Sino-German Cooperative Research Centre for Zoonosis of Animal Origin of Shandong Province, Shandong Provincial Key Laboratory of Animal Biotechnology and Disease Control and Prevention, Shandong Provincial Engineering Technology Research Center of Animal Disease Control and Prevention, Shandong Agricultural University, Tai’an City, China; ^2^Collaborative Innovation Center for the Origin and Control of Emerging Infectious Diseases, Taishan Medical University, Tai’an City, China

**Keywords:** rabbits, nucleotide-binding oligomerization domain 1, signaling pathway, autophagy, innate immunity, enterohemorrhagic *Escherichia coli*

## Abstract

Nucleotide-binding oligomerization domain 1 (NOD1) is the most prominent of all NOD-like receptors, which in the mammalian innate immune system, serve as intracellular receptors for pathogens and endogenous molecules during tissue injury. From rabbit kidney cells, we cloned rabbit NOD1 (rNOD1) and identified an N-terminal caspase activation and recruitment domain, a central NACHT domain, and C-terminal leucine-rich repeat domains. rNOD1 was expressed in all tested tissues; infection with *Escherichia coli* induced significantly higher expression in the spleen, liver, and kidney compared to other tissues. The overexpression of rNOD1 induced the expression of proinflammatory cytokines *Il1b, Il6, Il8, Ifn-γ*, and *Tnf* and defensins, including *Defb124, Defb125, Defb128, Defb135*, and *Np5 via* activation of the nuclear factor (NF)-κB pathway. Overexpression of rNOD1 inhibited the growth of *E. coli*, whereas knockdown of rNOD1 or inhibition of the NF-κB pathway promoted the growth of *E. coli*. rNOD1 colocalized with LC3, upregulated autophagy pathway protein LC3-II, and increased autolysosome formation in RK-13 cells infected with *E. coli*. In summary, our results explain the primary signaling pathway and antibacterial ability of rNOD1, as well as the induction of autophagy that it mediates. Such findings suggest that NOD1 could contribute to therapeutic strategies such as targets of new vaccine adjuvants or drugs.

## Introduction

Innate immunity, the first line of non-specific defense against infection, is mediated by phagocytes, such as macrophages and dendritic cells. A key aspect of these cells is the expression of pattern recognition receptors (PRRs), which have evolved to detect pathogen-associated molecular patterns (PAMPs) (1). To date, three primary families of PRRs have been described: toll-like receptors (TLRs), which are expressed on the cell surface and luminal side of intracellular vesicles (2, 3); nucleotide-binding oligomerization domain-like receptors (NLRs), which are located in the cytosol and function as cytoplasmic sensors (4, 5), and retinoic acid inducible gene-I-like receptors, which detect viruses (6).

As the best known family of PRRs, TLRs play important roles in host defense against microbial infections by sensing structural components of a variety of microbial pathogens, including bacteria, fungi, and viruses (7). Recognition of PAMPs by TLRs triggers the antimicrobial host defense responses and activates multiple steps in the inflammatory process by inducing type I-interferons (IFNs) and chemokines to eliminate invading pathogens (8). In mammals, the NLR family comprises large, multidomain proteins typically characterized by an N-terminal protein–protein interaction domain, a centrally located nucleotide oligomerization domain (NOD), also known as a NACHT domain, and C-terminal leucine-rich repeats (LRRs). The NACHT domain mediates self-regulation and oligomerization, whereas LRRs recognize pathogen-specific ligands (9–11). The N-terminal domain is responsible for activating downstream signaling through an amino-terminal effector-binding domain, which consists of caspase activation and recruitment domain (CARD), pyrin domain, acidic transactivator domain, or baculovirus inhibitor of apoptosis repeat domain. Depending on the composition of their N-terminal effector domains, NLRs are categorized into one of four subfamilies: NLRA, NLRB, NLRC, and NLRP (12). The NLRC subfamily has five members: NOD1, NOD2, NLRC3, NLRC4, and NLRC5, and participates in the initiation of proinflammatory responses by recognizing pathogens or microorganism as ligands (13, 14).

The best-characterized cytosolic sensor of the NLR family, NOD1, has received extensive attention and is well-studied in mammals, including humans (15), mice (15), and pigs (16). NOD1 can be activated by g-d-glutamyl-meso-diaminopimelic acid (iE-DAP), a motif that is present in the peptidoglycan of certain Gram-negative bacteria such as *Escherichia coli* (17), *Pseudomonas aeruginosa* (18), and *Campylobacter jejuni* (19), as well as some Gram-positive bacteria, including *Bacillus subtilis* (20) and *Listeria monocytogenes* (21). Recent research has linked bacterial sensing by NOD1 and NOD2 to the induction of autophagy and the formation of autophagosomes around invasive bacteria (22). NOD1, by extension, is expected to play an important role in sensing Gram-negative bacterial infections inside cells. Enterohemorrhagic *Escherichia coli* (EHEC) causes severe disease such as hemorrhagic colitis, hemolytic uremic syndrome, and attaching and effacing lesions, the last of which is a key virulence involved in EHEC’s colonization in the colon (23). EHEC is primarily transmitted to humans through contaminated food and water sources (24, 25), with the most common source of contaminated food being cattle. It has recently been confirmed that EHEC can be transmitted from cattle to wild rabbits (26, 27). Given the large wildlife rabbit population and the increasing use of rabbits for research and food production, the risk for further interspecies pathogen transmission is high (28).

After recognizing bacterial PAMPs, NOD proteins undergo conformational changes and self-oligomerize to recruit downstream effectors. The CARD of NOD1 binds with the CARD of either receptor-interacting protein 2 or receptor-interacting serine-threonine protein kinase-2, *via* CARD-CARD homophilic interactions. This interaction stimulates nuclear factor (NF)-κB by inhibiting the NF-κB kinase complex and induces the production of proinflammatory cytokine *Il1b* and the recruitment of immune cells, including neutrophils and macrophages (29). NOD signaling also activates the mitogen-activated protein kinase (MAPK) pathway, which stimulates the activation of specific transcription factors such as activator protein-1, thereby inducing the production of proinflammatory cytokines and chemokines, such as IL-1β, IL-6, IL-8, IL-12, TNF-α, and IFN-γ (11, 30–33).

Recent studies have shown that the same innate immune factors recognize different microbial components and mediate different immune responses in different species. Murine TLR7 and TLR9 favor sequence-specific motifs that are distinct from those recognized by human TLR7 and TLR9 (34, 35). TLR8, which recognizes GU-rich ssRNA in humans, is nonfunctional in mice (36). Stimulator of interferon genes (STING) is a key component of the RIG-I pathway, but not the melanoma differentiation-associated protein 5 (MDA5) pathway in mammals. However, it can activate the MDA5-STING-IFN-β pathway in chickens (37). Human macrophages sense all bacterial RNA components and synthetic ssRNA to activate the NLRP3 inflammasome, whereas murine macrophages preferentially recognize bacterial mRNA (38). Thus, rabbit NOD1 (rNOD1) may mediate different immune responses in humans and mice. Although the predicted gene sequence of rNOD1 is known (NCBI XM_008261590.2), it has not been studied experimentally. We aimed to elucidate rNOD1-mediated signaling pathway and determined whether it has a similar role in NF-κB signaling pathway as NOD1 in human and mouse, and whether it exhibits antimicrobial activity. To this end, we cloned and characterized rNOD1 and investigated its downstream signaling pathways and antibacterial activity to clarify its role during bacterial infections.

## Materials and Methods

### Reagents, Cells, Bacteria, and Animals

SP600125 (a JNK inhibitor), SB203580 (a p38 MAPK inhibitor), U0126 (an ERK inhibitor), and BAY11-7082 (an NF-κB inhibitor) were obtained from MedChem Express (Monmouth Junction, NJ, USA). C12-iE-DAP was obtained from InvivoGen (San Diego, CA, USA).

Rabbit kidney cells (RK-13) were cultured and maintained in Dulbecco’s modified Eagle medium (Gibco, Grand Island, MI, USA) containing 10% fetal bovine serum (TransGen, Beijing, China) at 37°C in 5% (v/v) CO_2_.

The bacterial pathogen EHEC was originally isolated from clinically infected rabbits suffering from acute diarrhea, and was stored at the Environmental Microbiology Laboratory at Shandong Agricultural University.

Healthy, weaned, 35-day-old New Zealand White rabbits were raised in the same environment with sufficient room, food, and ventilation for each rabbit.

### Cloning and Analysis of the rNOD1 Sequence

Total RNA was extracted from the RK-13 cells using *Trans*Zol (TransGen) and first cDNA synthesis was carried out using HiScript^®^ II Q Select RT SuperMix for qPCR (+gDNA wiper) (Vazyme, Nanjing, China). To clone rNOD1, primers were designed based on the predicated gene sequence published in GenBank (Table 1). The amino acid sequence of rNOD1 was aligned with that of other species using Clustalx. The CARD, NACHT, and LRR regions of rNOD1 were analyzed using the sample modular architecture research tool (SMART). Lastly, phylogenetic analysis of rNOD1 was conducted using the neighbor-joining method of the MEGA5.1 program with 1,000 bootstrap replications to validate the branches.

**Table 1:** Primers used in this study.

Primer name	Sequence (5′–3′)	Purpose
rNOD1 F	cgaagatgaattcgggagaa	Gene cloning
rNOD1 R	ggcttcgaacaaaagcagtt	
rNOD1-CARD F	tccactagtccagtgtggtggaattcgccaccatggtcaagctgctgaaaatcca	Gene cloning
rNOD1-CARD R	gggtagaaaggtcctctagactcgag cagccacggcctgaggtcca	
rNOD1-delCARD F	tccactagtccagtgtggtggaattcgccaccatgtcggacgtgggcttcatc	Gene cloning
rNOD1-delCARD R	gggtagaaaggtcctctagactcgaggaaacagatgagcctctt	
qdrNOD1 F	acaaggttcgcaaaatcctg	RT-PCR
qdrNOD1 R	ttgacaatggctttgctctg	
*Il1b* F	tggcacgtatgagctgaaag	RT-PCR
*Il1b* R	ggccacaggtatcttgtcgt	
*Il4* F	cactccggcagttctacctc	RT-PCR
*Il4* R	gcagaggttcctgtcgagtc	
*Il6* F	ctgaagacgaccacgatcca	RT-PCR
*Il6* R	aaggacacccgcactccat	
*Il8* F	ctctcttggcaaccttcctg	RT-PCR
*Il8* R	ttgcacagtgaggtccactc	
*Il10* F	aaaagctaaaagccccagga	RT-PCR
*Il10* R	cgggagctgaggtatcagag	
*Ifn-γ* F	ctcgaatttcggtggatgat	RT-PCR
*Ifn-γ* R	agcgtctgactcctttttcg	
*Tnf* F	cacttcagggtgatcggc	RT-PCR
*Tnf* R	tgcgggtttgctactacg	
*Defb124* F	gcaccaagcaagagtccttc	RT-PCR
*Defb124* R	acgccagagccagctactta	
*Defb125* F	cgtgctgcatctccttaaca	RT-PCR
*Defb125* R	gcgaagcagaaaattgatcc	
*Defb128* F	gggctcaaggctttctcttt	RT-PCR
*Defb128* R	aaatctcgcctagcttgcac	
*Defb135* F	gctgcatctccaaatccaat	RT-PCR
*Defb135* R	tagtgggatggtgcaactga	
*Np5* F	aggcaggcgtgttctgtact	RT-PCR
*Np5* R	ggtctccacgcaaataagga	
*Gapdh* F	aggtcatccacgaccacttc	RT-PCR
*Gapdh* R	Gtgagtttcccgttcagctc	

### Expression of rNOD1 *In Vivo*

Quantitative real-time PCR (qRT-PCR) was used to determine the expression of rNOD1 in various tissues. Five healthy rabbits were euthanized and the heart, liver, spleen, lungs, kidneys, trachea, thymus, esophagus, stomach, duodenum, jejunum, ileum, cecum, colon, rectum, pancreas, appendix, mesenteric lymph nodes, lymph follicles, sacculus rotundus, brain, cerebellum, brainstem, muscles, and skin were collected. Total RNA was isolated from each sample and cDNA was prepared as described above. The primers used to evaluate the expression of rNOD1 are shown in Table 1; glyceraldehyde-3-phosphate-dehydrogenase (*Gapdh*) was used as an internal control.

Rabbits were infected with 10^8^ CFU of *E. coli* bacterial suspension by intraperitoneal injections. At 1, 2, and 3 days postinfection (dpi), five rabbits of each group were euthanized and the liver, spleen, and kidney were collected for RNA extraction. All animal experiments were conducted in duplicate.

### Construction of Recombinant Expression Vectors

The following sequences were amplified using the primers shown in Table 1: the full length rNOD1, rNOD1-CARD, and rNOD1 without CARD (rNOD1-delCARD). All fragments were cloned into the pCDNA3.1 (+) vector using the Hieff Clone™ Multi One Step Cloning Kit (Yeasen, Shanghai, China) to make the constructs pC-rNOD1, pC-rNOD1-CARD, and pC-rNOD1-delCARD. pCDNA3.1-empty vector was used as the control. RK-13 cells were plated in six-well plates for 12 h prior to transfection. 2 µg of pC-rNOD1, pC-rNOD1-CARD, pC-rNOD1-delCARD, or pCDNA3.1-empty were transfected using *Trans*IL-LT1 Transfection Reagent (MirusBio, Madison, WI, USA) for 24 h, after which the cells were harvested for RNA extraction.

### siRNA Interference

1 µg of siRNA or negative control (NC) siRNA were transfected with *Trans*IL-LT1 Transfection Reagent. The si-RNA sequences used were: si-rNOD1-1, sense 5′-GGAUCAUCUUUCGCUGCUUTT-3′, antisense 5′-AAGCAGCGAAAGAUGAUCCTT-3′; si-rNOD1-2, sense 5′-GCAUGUUCAGCUGCUUCAATT-3′, antisense 5′-UUGAAGCAGCUGAACAUGCTT-3′; si-rNOD1-3, sense 5′-CCAAGAGCCUGUUUGUCUUTT-3′, antisense 5′-AAGACAAACAGGCUCUUGGTT-3′; NC, sense 5′-UUCUCCGAACGUGUCACGUTT-3′, antisense 5′-ACGUGACACGUUCGGAGAATT-3′. siRNA sequences were synthesized by GenePharma (Shanghai, China).

### Luciferase Assays

RK-13 cells were plated in 24-well plates for 12 h prior to transfection. The luciferase reporter plasmids pGL3-NF-κB, pGL3-IFN-β, and pGL3-ISRE were purchased from Agilent (Santa Clara, CA, USA). The pRL-TK plasmid (Promega, Madison, WI, USA) acted as an internal control to normalize transfection efficiency. Cells were transfected with reporter plasmid (100 ng/well) or pRL-TK plasmid (50 ng/well) and 1 µg of pC-rNOD1, pC-rNOD1-CARD, pC-rNOD1-delCARD, or pCDNA3.1-empty or with 500 ng of si-rNOD1 or NC siRNA for 24 h using *Trans*IL-LT1 Transfection Reagent. After 24 h of cotransfection with NF-κB, the cells were infected with 1 × 10^7^ *E. coli* for 2 h. The medium was then removed, and the cells were cultured in DMEM containing gentamicin (100 µg/mL) for 3 h. After lysing and harvesting cells, luciferase activities were detected with a dual-luciferase reporter assay system (Beyotime, Wuhan, China).

### *E. coli* Infection

RK-13 cells were plated in 24-well plates for 12 h prior to transfection. 1 µg of pC-rNOD1 or pCDNA3.1-empty or 500 ng of si-rNOD1 or NC siRNA were transfected into cells for 24 h using *Trans*IL-LT1 Transfection Reagent. The cells were then infected with 1 × 10^7^ *E. coli* for 2 h, the medium was removed, and cells were washed three times with PBS containing gentamicin (100 µg/mL) to eliminate extracellular *E. coli*. The cells were then cultured in DMEM containing gentamicin for 3 h. RK-13 cells were lysed in 500 µL PBS containing 1% (v/v) Triton X-100 for 20 min and were then plated onto nutrient agar to calculate the intracellular bacterial CFU.

### qRT-PCR Analysis

Total RNA was extracted from the liver, spleen, and kidney of rabbits, as well as from RK-13 cells, using the RNeasy Plus Mini Kit (Qiagen, Hilden, Germany) according to the manufacturer’s instructions. Total RNA (1 µg) was reverse transcribed using TransScriptR One-step gDNA Removal and cDNA Synthesis SuperMix for qPCR (Transgen Biotech Co., Ltd., Beijing, China). The synthesized cDNA was stored at −20°C until further use. qRT-PCR was performed using TransStart^®^ Tip Green qPCR SuperMix (+Dye II) (Transgen Biotech). qRT-PCR primers were designed using the Primer 3 software (http://bioinfo.ut.ee/primer3-0.4.0/) based on published target sequences (Table 1). The primers *Il6* and *Tnf* used for qRT-PCR have been previously reported (39). qRT-PCR was carried out using a 7500 Fast Real-Time PCR system (Applied Biosystems, Carlsbad, CA, USA). The PCR conditions were as follows: 1 cycle at 94°C for 30 s, 40 cycles at 94°C for 5 s, and 60°C for 34 s. Dissociation curves analysis was performed as the final step of the PCR.

### Confocal Immunofluorescence Microscopy

RK-13 cells were seeded on sterile glass cover slips placed in 24-well plates and cotransfected with pC-rNOD1 and GFP-LC3 plasmids for 24 h. The cells were then infected with 1 × 10^7^ *E. coli* for 2 h, the medium was removed, and cells were washed three times with PBS containing gentamicin (100 µg/mL) to eliminate extracellular *E. coli*. Cells were then cultured in DMEM containing gentamicin for 3 h, washed with PBS, fixed with 4% paraformaldehyde for 8 min, and treated with 0.1% Triton X-100 for 10 min. Cells were blocked in PBS containing 5% bovine serum albumin for 30 min, incubated with primary antibodies for 1 h at 37°C, and then incubated for 1 h with secondary antibody of Cy3-labeled goat antimouse IgG. Images were captured using a Leica TCS SPE confocal microscope with a 63× (1.3 numerical aperture) oil immersion objective.

### Western Blotting Analysis

Total protein lysates were obtained by lysing the cells with ice-cold RIPA buffer supplemented with a protease inhibitor cocktail (Beyotime). Protein was quantified using a BCA protein assay kit (Tiangen, Beijing, China), samples were run on a SDS-PAGE and transferred to PVDF membranes. After blocking with 5% skim milk for 1 h at room temperature, membranes were incubated overnight at 4°C with the primary antibodies, and then incubated with appropriate secondary antibodies for 2 h at room temperature. Images of protein blots were obtained with a ChemiDoc XRS (Bio-Rad, Marnes-la-Coquette, France) using a Western ECL Substrate kit. The density of each band was normalized to that of β-actin and quantified it using Quantity One software (Bio-Rad).

### Statistical Analyses

The relative expression of each gene was calculated using the 2^−ΔΔCt^ method. The housekeeping gene *Gapdh* was used as an endogenous control to normalize the expression of target genes. Each treatment was conducted in triplicate and each *in vitro* assay was performed in triplicate. The data were analyzed using the non-parametric Mann–Whitney *U* test. Statistical analyses were performed using GraphPad Prism 5.0 (GraphPad Software Inc., San Diego, CA, USA). Statistical significance was set at *P* < 0.05.

## Results

### Sequence Analysis of rNOD1

**Figure 1:**
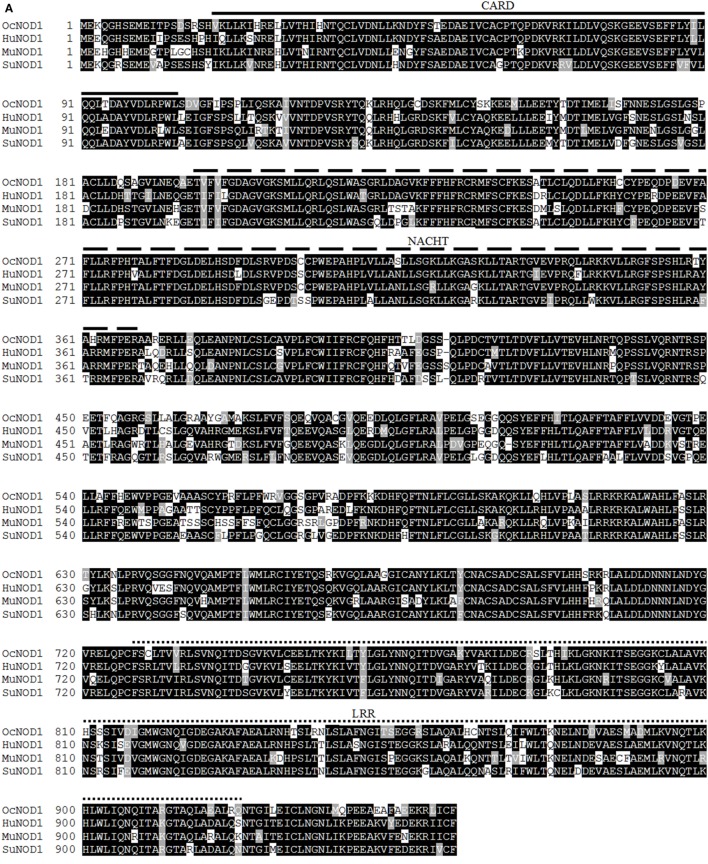

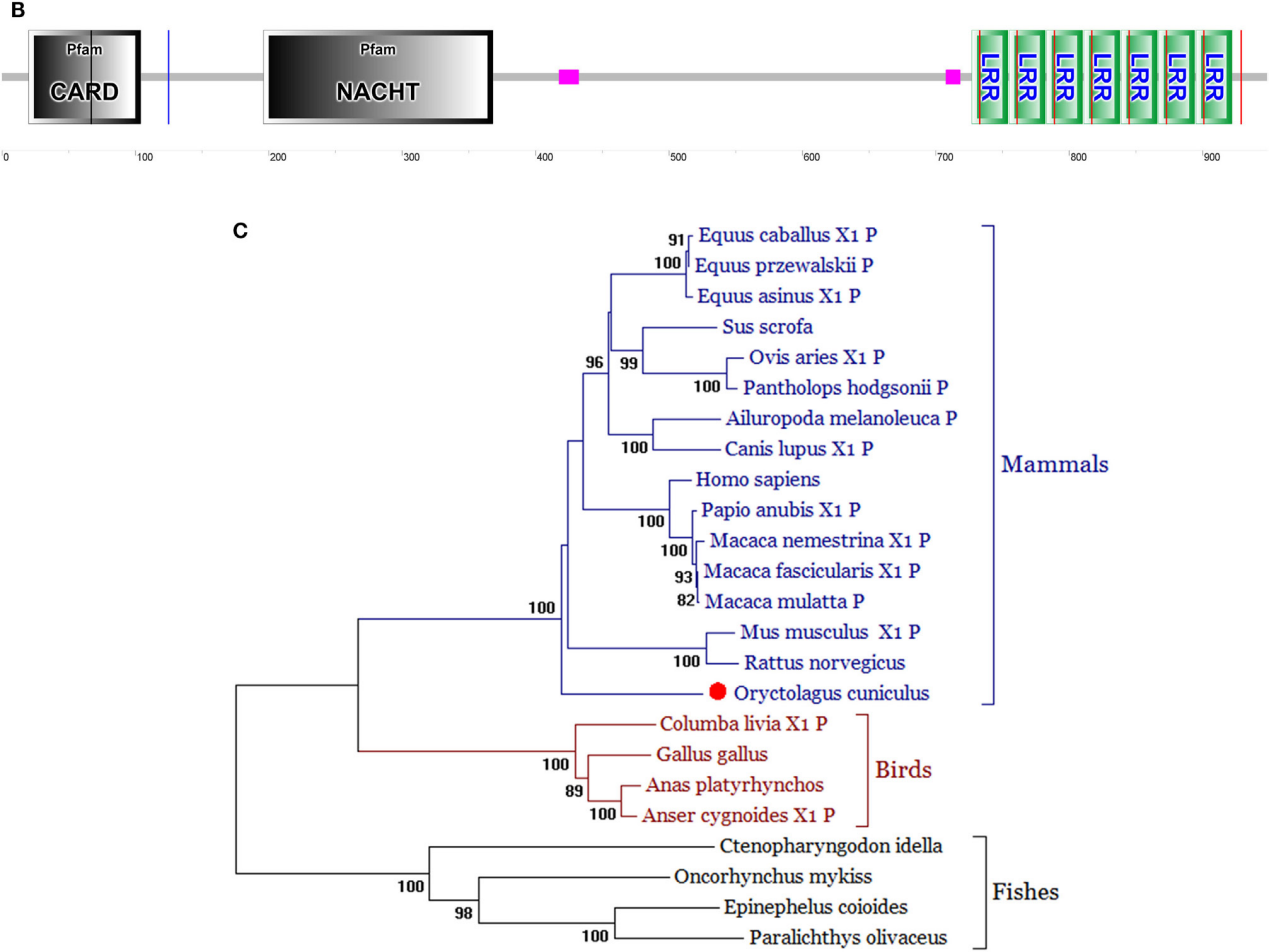
Sequence analysis of rabbit nucleotide-binding oligomerization domain 1 (rNOD1). **(A)** Alignment of amino acid sequence in *Oryctolagus curiculus* (MF069503), *Homo sapiens* (NP_006083.1), *Mus musculus* (NP_ 766317.1), and *Sus scrofa* (NP_001107749.1) NOD1. Alignment was performed using Clustal X and edited with Boxshade. Black shading indicates amino acid identity; gray shading indicates similarity (50% threshold). The caspase activation and recruitment domain (CARD), NACHT, and leucine-rich repeat (LRR) domains were indicated in this figure. **(B)** Protein motifs of rNOD1 were analyzed using sample modular architecture research tool (SMART). **(C)** A phylogenic tree based on rNOD1 between *O. curiculus* and other species. A neighbor-joining tree was generated using MEGA 5.0, and a 1,000-bootstrap analysis was performed. The sequences used were: *Equus caballus* NOD1, XP_001499616.1; *Equus przewalskii* NOD1, XP_008521180.1; *Equus asinus* NOD1, XP_014696539.1; *Sus scrofa* NOD1, NP_001107749.1; *Ovis aries* NOD1, XP_ 004007979.1; *Pantholops hodgsonii* NOD1, XP_005981278.1; *Ailuropoda melanoleuca* NOD1, XP_002919315.1; *Canis lupus* NOD1, XP_013974566.1; *Homo sapiens* NOD1, NP_006083.1; *Papio anubis* NOD1, XP_003896196.1; *Macaca nemestrina* NOD1, XP_011729625.1; *Macaca fascicularis* NOD1, XP_005549941.1; *Macaca mulatta* NOD1, XP_001085719.1; *Mus musculus* NOD1, NP_ 766317.1; *Rattus norvegicus* NOD1, NP_001102706.1; Oryctolagus curiculus NOD1, MF069503; *Columba livia* NOD1, XP_005510067.1; *Gallus gallus* NOD1, NP_001305367.1; *Anas platyrhynchos* NOD1, NP_001297310.1; *Anser cygnoides* NOD1, XP_013053223.1; *Ctenopharyngodon idella* NOD1, ACX71752.1; *Oncorhynchus mykiss* NOD1, AII73558.1; *Epinephelus coioides* NOD1, AFV53357.1; *Paralichthys olivaceus* NOD1, AFD29894.1.

The complete open reading frame of rNOD1 was obtained and the sequence was submitted to GenBank (MF069503). The sequence contained 2,862 bp encoding 953 amino acids. Secondary structures were predicated using SMART, the results of which indicated that rNOD1 contained three characteristic domains: an N-terminal CARD (aa 20–104), a central NACHT domain (aa 196–368), and C-terminal LRR domains (aa 727–754, 755–782, 783–810, 811–838, 839–866, 867–894, and 895–922), all shown in Figures 1A,B.

The deduced amino acid sequence of rNOD1 was 81.7, 79.2, and 80.7% identical to those of *Homo sapiens, Mus musculus*, and *Sus scrofa*, respectively (Table 2). The rNOD1 NACHT domain was closest in sequence identity to that of other species (Table 2). A phylogenetic tree was constructed with the full length NOD1 protein and three major branches were observed (Figure 1C). Among the mammalian species, *Rattus norvegicus* showed the closest evolutionary relationship with rNOD1.

**Table 2:** Amino acid identity (%) of rNOD1 and its domains (CARD, NACHT, and LRRs) compared to other species.

Species	Full length	CARD	NACHT	LRRs
*Homo sapiens*	81.7	90.6	90.2	81.1
*Mus musculus*	79.2	85.9	91.3	78.6
*Sus scrofa*	80.7	83.5	86.7	81.1

### Expression of rNOD1 *In Vivo*

**Figure 2:**
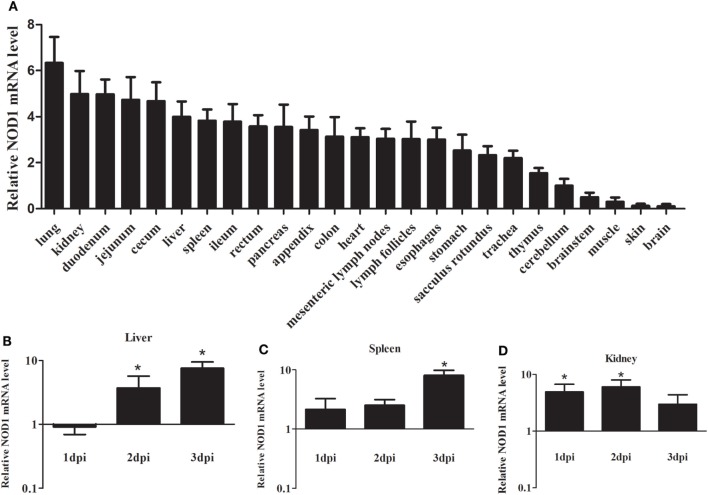
Expression profiles of rabbit nucleotide-binding oligomerization domain 1 (rNOD1) in rabbits. **(A)** Expression of rNOD1 in tissues of healthy rabbits. Expression of rNOD1 in the **(B)** liver, **(C)** spleen, and **(D)** kidney of rabbits infected with *Escherichia coli* (10^8^ CFU). The relative expressions of rNOD1 were normalized to *Gapdh*. *M* ± SD from two independent repetitions are presented. Significant differences are indicated by *.

Quantitative real-time PCR was performed to analyze the expression levels of rNOD1 mRNA in tissues of healthy rabbits. Although rNOD1 was detected in all tested tissues, higher levels of expression were detected in lung, kidney, duodenum, jejunum, cecum, liver, spleen, ileum, rectum, pancreas, appendix, colon, heart, mesenteric lymph nodes, lymph follicles, esophagus, stomach, sacculus rotundus, trachea, and thymus than in cerebellum, brainstem, muscle, skin, and brain (Figure 2A).

To evaluate the response of rNOD1 expression to bacterial infection, we infected rabbits with *E. coli* and examined rNOD1 mRNA transcripts in liver, spleen, and kidney at 1, 2, and 3 dpi using qRT-PCR. As shown in Figure 2B, although the expression of rNOD1 displayed no significant difference at 1 dpi, it became significantly upregulated at 2 dpi by 3.69-fold (*P* < 0.05) and 3 dpi by 7.68-fold (*P* < 0.05) in the liver, whereas the expression of rNOD1 displayed significantly upregulated at 3 dpi by 8.01-fold (*P* < 0.05) in the spleen (Figure 2C). Significant upregulation of rNOD1 mRNA expression was detected in kidney at 1 dpi (4.88-fold) and 2 dpi (5.98-fold) (Figure 2D).

### rNOD1 Signaling through the NF-κB Pathway

**Figure 3:**
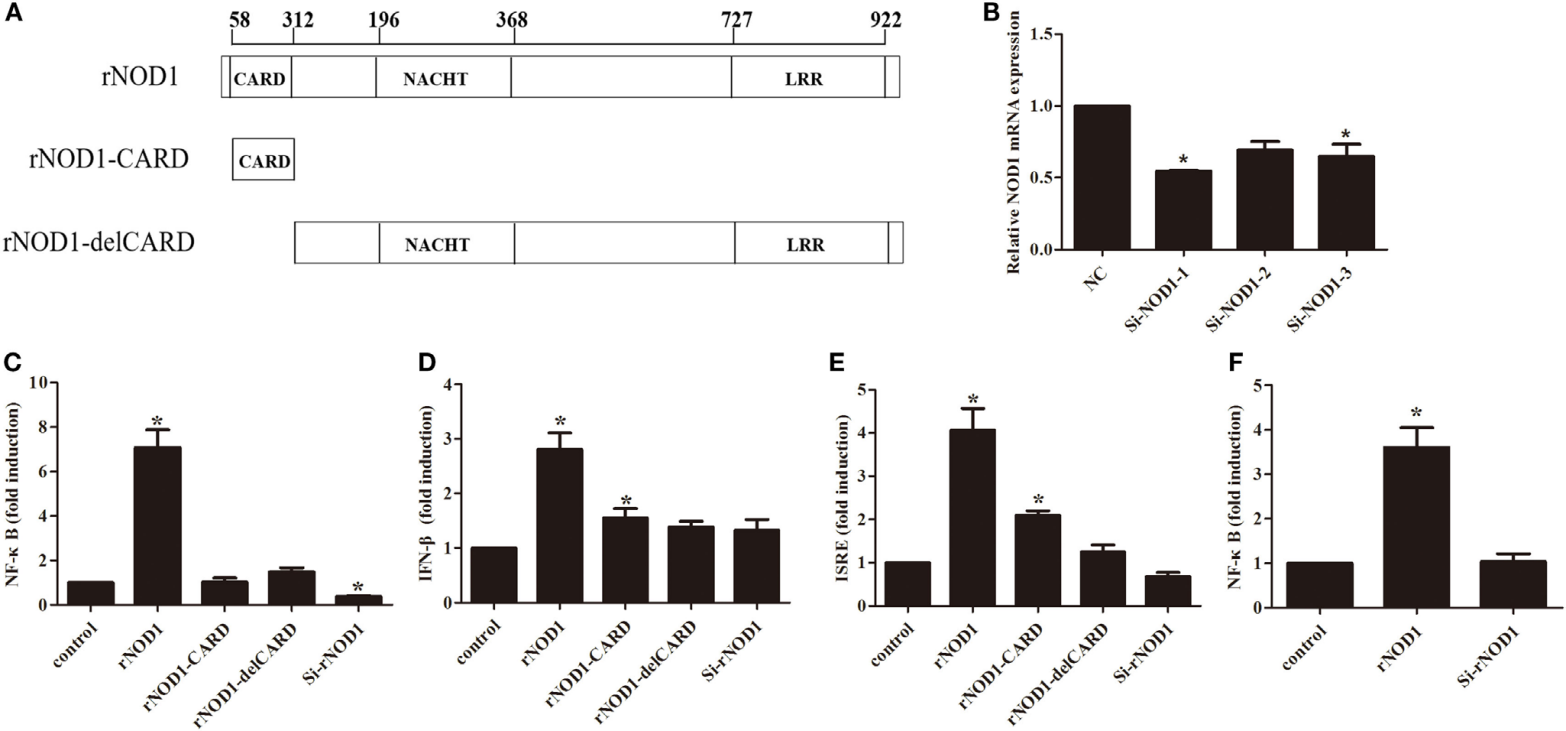
Effects of nuclear factor-κB (NF-κB), interferon (IFN-β), and ISRE on different rabbit nucleotide-binding oligomerization domain 1 (rNOD1) domains and si-NOD1. **(A)** Domains used to construct pC-rNOD1, pC-rNOD1-CARD, and pC-rNOD1-delCARD plasmids. **(B)** Silencing efficiency of siRNA targeting rNOD1. RK-13 cells were transfected with siRNA1, 2, or 3 for 24 h and NOD1 expression was analyzed. NC siRNA was used as a control. *Gapdh* was used as the internal control. pC-rNOD1, pC-rNOD1-CARD, pC-rNOD1-delCARD, or pCDNA3.1-empty or with si-rNOD1 or NC siRNA with **(C)** NF-κB, **(D)** IFN-β, and **(E)** ISRE reporter plasmid, and pRL-TK plasmid were transfected into cells for 24 h. **(F)** After 24 h of cotransfection with NF-κB, the cells were infected with *Escherichia coli*. *M* ± SD from three independent experiments are presented. Significant differences are indicated by *.

To evaluate the role of rNOD1 in NF-κB signaling and to clarify which domain of rNOD1 mediates this role, mutant plasmids were constructed (Figure 3A). In addition, three siRNAs (si-rNOD1-1, si-rNOD1-2, and si-rNOD1-3) were designed to target different regions of rNOD1. The knockdown efficiency of si-rNOD1-1 was 54.57% (Figure 3B).

As shown in Figure 3C, overexpression of rNOD1 significantly increased NF-κB activity by 33.54-fold (*P* < 0.05) compared to that induced by the empty vector. rNOD1-CARD and del-CARD mutants were unable to significantly increase NF-κB activity, even after 24 h (Figure 3C). Addition of rNOD1-specific siRNA reduced NF-κB activity (0.38-fold, *P* < 0.05), and rNOD1 increased IFN-β and ISRE activity by 2.80- and 4.07-fold, respectively. By contrast, addition of si-rNOD1 did not affect basal IFN-β or ISRE activity (Figures 3D,E). Finally, to evaluate the effect of rNOD1 on *E. coli*-induced NF-κB activity, RK-13 cells were infected with *E. coli* after transfected with rNOD1. rNOD1 significantly increased NF-κB activity by 3.60-fold (*P* < 0.05) when induced by *E. coli* (Figure 3F).

### Induction of Cytokines and Defensins by rNOD1 Effector Domains

**Figure 4:**
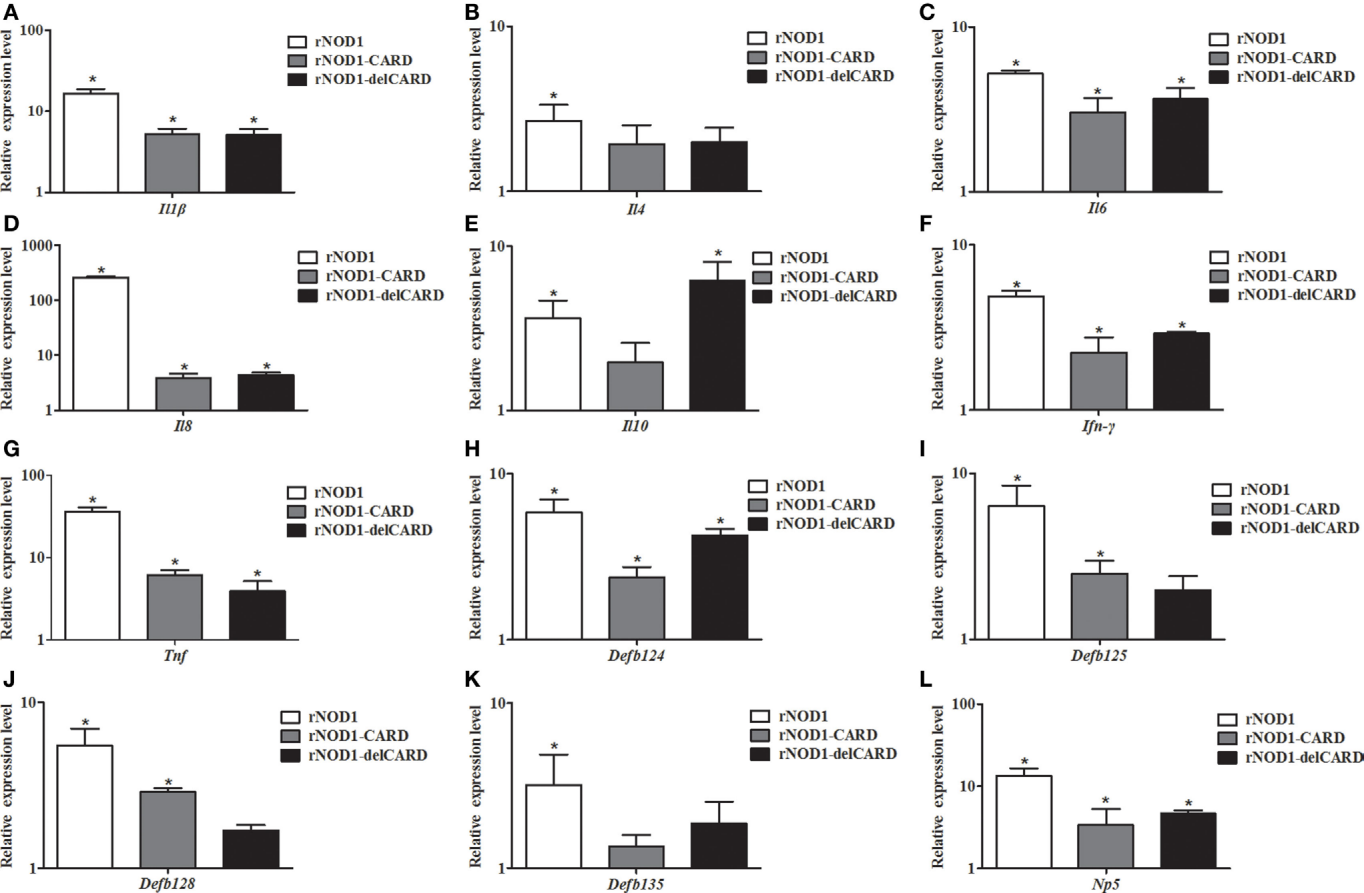
Expression profiles of immune-related genes in RK-13 cells. Two microgram of pC-rNOD1, pC-rNOD1-CARD, pC-rNOD1-delCARD, and pCDNA3.1-empty were transfected for 24 h. The Effects of the overexpression of different rNOD1 domains on expression of **(A)** *Il1b*, **(B)** *Il4*, **(C)** *Il6*, **(D)** *Il8*, **(E)** *Il10*, **(F)** *Ifn-γ*, **(G)** *Tnf*, **(H)** *Defb124*, **(I)** *Defb125*, **(J)** *Defb128*, **(K)** *Defb135*, and **(L)** *Np5* were determined. *M* ± SD from three independent experiments are presented. Significant differences are indicated by *.

To investigate the induction of innate immune response by rNOD1 effector domains in RK-13 cells, the expression of cytokines and defensins was analyzed *via* qRT-PCR. The expressions of proinflammatory cytokines *Il1b, Il6, Il8, Ifn-γ*, and *Tnf* became significantly up-regulated with the overexpression of rNOD1 effector domains (Figures 4A,C,D,F,G). A 258.71-fold (*P* < 0.05) increase in *Il8* expression was detected after transfection with rNOD1 (Figure 4D). The anti-inflammatory cytokine *Il4* became significantly upregulated by only 2.67-fold (*P* < 0.05; Figure 4B) with the overexpression of rNOD1, whereas *Il10* became upregulated by 3.67- and 6.20-fold (*P* < 0.05; Figure 4E) with overexpression of rNOD1 and del-CARD domains, respectively. Overexpression of full length rNOD1 significantly increased the expression of α-defensin (*Np5*) and β-defensins (*Defb124, Defb125, Defb128*, and *Defb135*) compared to overexpression of the empty vector (Figures 4H–L). Induction of α- and β-defensins by rNOD1-CARD and del-CARD mutants was significantly less than that of full length rNOD1. In particular, rNOD1 overexpression significantly increased expression of *Defb125* by 6.39-fold; by contrast, overexpression of rNOD1-CARD and del-CARD mutants increased expression of *Defb125* by 2.50-fold (*P* < 0.05) and 1.98-fold (*P* > 0.05), respectively.

### Antibacterial Activity of rNOD1

**Figure 5:**
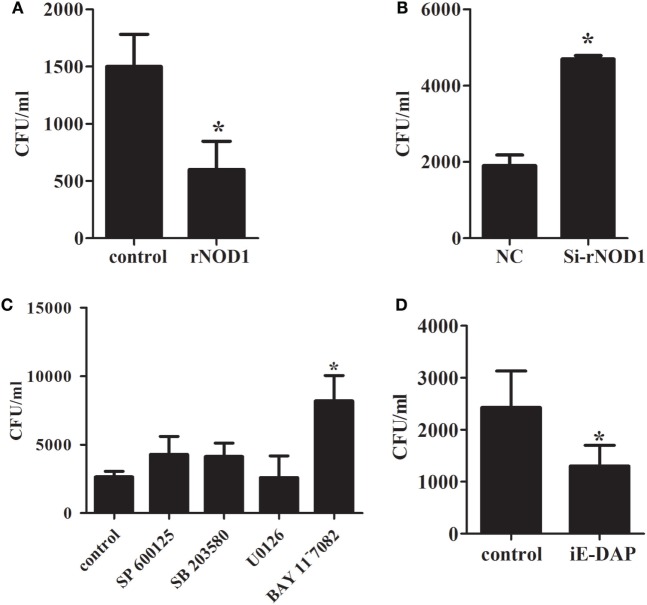
Effect of antimicrobial activity with the overexpression or knockdown of rabbit nucleotide-binding oligomerization domain 1 (rNOD1). **(A)** pC-rNOD1 and **(B)** si-rNOD1 were transfected into RK-13 cells for 24 h. pCDNA3.1-empty and negative control (NC) siRNA were used as controls, respectively. **(C)** After 24 h of transfection, cells were treated with 10 µM of SP600125, SB203580, U0126, or BAY11^−^7082 for 12 h. **(D)** 1 µg/mL of iE-DAP were transfected into cells for 12 h. Cells were infected with 1 × 10^7^ *Escherichia coli*. *M* ± *SD* from three independent experiments are presented. Significant differences are indicated by *.

To investigate the ability of rNOD1 to regulate an antimicrobial response to *E. coli*, RK-13 cells were infected with *E. coli* after transfection with pC-rNOD1 or empty vector and si-rNOD1 or NC siRNA. As shown in Figure 5A, RK-13 cells transfected with rNOD1 contained significantly less *E. coli* CFUs than those transfected with the empty vector. By contrast, cells transfected with si-rNOD1 contained significantly more *E. coli* CFUs than those transfected with NC siRNA (Figure 5B). To further investigate which pathway was activated by rNOD1, cells were pretreated with inhibitors of NF-κB activity and three major components of the MAPK pathway. Inhibition of the NF-κB pathway, but not the MAPK pathway eliminated the ability for rNOD1 to inhibit *E. coli* growth (Figure 5C). As shown in Figure 5D, the number of *E. coli* was inhibited after stimulated with iE-DAP.

### Expression of Immune-Related Genes in RK-13 Cells Infected with *E. coli*

**Figure 6:**
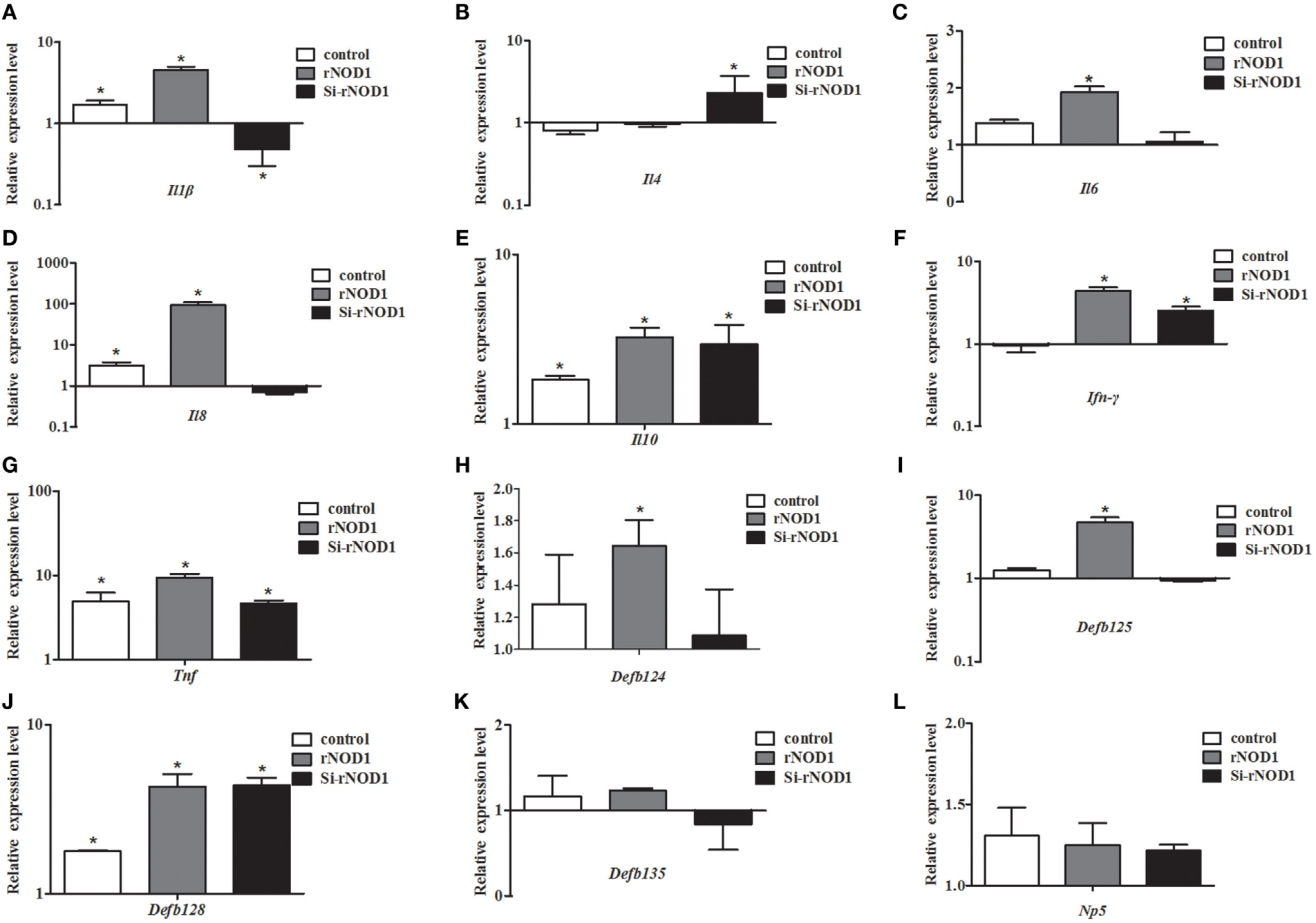
Effect of the overexpression or knockdown of rabbit nucleotide-binding oligomerization domain 1 (rNOD1) on expression of immune-related genes induced by *Escherichia coli*. Expression of **(A)** *Il1b*, **(B)** *Il4*, **(C)** *Il6*, **(D)** *Il8*, **(E)** *Il10*, **(F)** *Ifn-γ*, **(G)** *Tnf*, **(H)** *Defb124*, **(I)** *Defb125*, **(J)** *Defb128*, **(K)** *Defb135*, and **(L)** *Np5* in *E. coli-*infected RK-13 cells transfected with pC-rNOD1 or si-rNOD1. *M* ± SD from three independent experiments are presented. Significant differences are indicated by *.

The effect of rNOD1 overexpression or knockdown on *E. coli*-induced cytokine and defensin production was evaluated. Overexpression of rNOD1 increased *E. coli*-induced proinflammatory cytokines *Il1b, Il6, Il8, Ifn-γ*, and *Tnf* (Figures 6A,C,D,F,G). Knockdown of rNOD1 significantly reduced *E. coli*-induced proinflammatory cytokine expression, but significantly increased the expression of anti-inflammatory cytokines *Il4* by 2.32-fold (*P* < 0.05; Figure 6B) and *Il10* by 2.96-fold (*P* < 0.05; Figure 6E). Similarly, overexpression of rNOD1 increased *E. coli*-induced *Defb124, Defb 125*, and *Defb128* expression, whereas knockdown of rNOD1 impaired the expression of these defensins (Figures 6H–J). No significant changes were detected in the expression of *Defb135* and *Np5* (Figures 6K,L).

**Figure 7:**
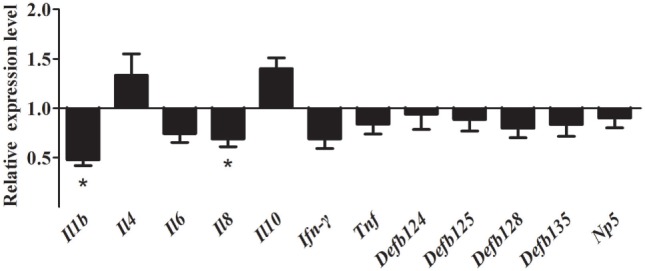
Effect of the overexpression of rabbit nucleotide-binding oligomerization domain 1 (rNOD1) on the expression of immune-related genes induced by *Escherichia coli* after inhibition of nuclear factor (NF)-κB. pC-rNOD1 or pCDNA3.1-empty were transfected into cells for 24 h. Cells were pretreated with 10 µM of BAY11^-^7082 for 12 h to inhibit NF-κB pathways and infected with *E. coli. M* ± SD from three independent experiments are presented. Significant differences are indicated by *.

After inhibition of the NF-κB pathway, overexpression of rNOD1 decreased the expression of proinflammatory cytokines and defensins induced by *E. coli*. In particular, the expression of *Il1b* and *Il8* were significantly decreased to 0.47- and 0.65-fold, respectively (*P* < 0.05). Although the expression of all defensins was reduced, no changes were statistically significant. By contrast, the expression of anti-inflammatory cytokines *Il4* and *Il10* was increased (Figure 7).

**Figure 8:**
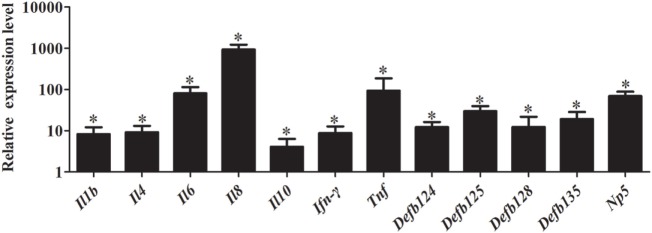
Effect of iE-DAP on expression of immune-related genes induced by *Escherichia coli*. RK-13 cells were treated with1μg/mL of iE-DAP for 12 h and then were infected with *E. coli*. *M* ± SD from three independent experiments are presented. Significant differences are indicated by *.

As shown in Figure 8, stimulation with iE-DAP significantly induced the expression of immune-related genes in RK-13 cells after *E. coli* infection. Especially, the expression of *Il6, Il8*, and *Tnf* were significantly upregulated by 81.20-fold, 940.00-fold, and 93.80-fold, respectively (*P* < 0.05). Similarly, expression of defensins *Defb125* (29.80-fold), *Defb135* (19.18-fold), and *Np5* (69.69-fold) was significantly increased.

### rNOD1 Induces Autophagosome Formation in RK-13 Cells Infected with *E. coli*

**Figure 9:**
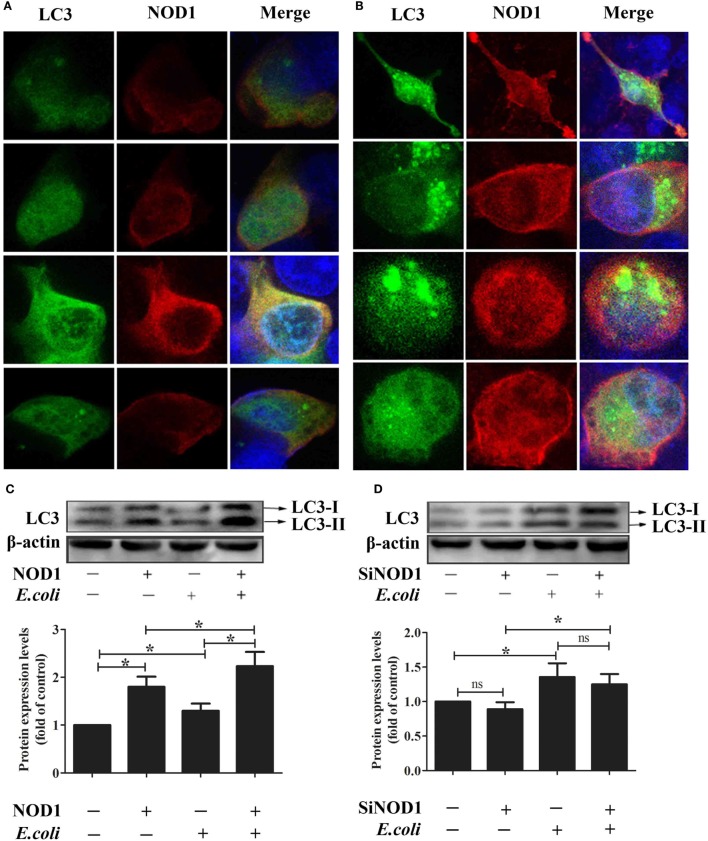
Rabbit nucleotide-binding oligomerization domain 1 (rNOD1) induces autophagosome formation in RK-13 cells. **(A)** Immunofluorescent imaging of RK-13 cells cotransfected with pC-rNOD1 and GFP-LC3 plasmids. rNOD1 appears in red, LC3 in green, and DAPI in blue. **(B)** Immunofluorescent imaging of RK-13 cells cotransfected with pC-rNOD1 and GFP-LC3 plasmids and infected with *E. coli*. rNOD1 appears in red, LC3 in green, and DAPI in blue. Immunoblot analysis of RK-13 cells transfected with **(C)** pC-rNOD1 or **(D)** si-rNOD1, followed by infection with *E. coli*. Data are expressed as the ratio of protein band intensity in treated cells to that of the corresponding protein in control cells. LC3-II expression was normalized to β-actin expression. *M* ± SD from three independent experiments are presented. Significant differences are indicated by *.

To investigate the ability for rNOD1 to induce autophagy, the colocalization of LC3 with rNOD1 was first evaluated. LC3 and rNOD1 colocalized in RK-13 cells (Figures 9A,B), and *E. coli* significantly induced autophagosome formation (Figure 9B), as similar phenomena shown in Figures 9C,D. Overexpression of rNOD1 significantly increased the protein levels of LC3-II in RK-13 cells infected with *E. coli* (Figure 9C). A slight decrease in the protein level of LC3-II was measured after knockdown of rNOD1 (Figure 9D).

## Discussion

Although NOD1 has been cloned from certain mammals and fish (33), rNOD1 had not yet been studied. Here, we cloned the rNOD1 gene from RK-13 cells and showed that it contains a CARD at the N-terminal region, seven LRRs at the C-terminal region, and a NACHT domain between the N- and C-termini. The rNOD1 protein shared 81.7% sequence identity with the humans, 79.2% identity with *M. musculus*, and 80.7% identity with *S. scrofa*. The NACHT domain was the most homologous domain between rabbits and other species, suggesting that NACHT is an evolutionarily conserved protein domain. Phylogenetic tree analyses showed that rNOD1 has a high degree of similarity with the mammal NOD1, especially *R. norvegicus*.

Previous studies have reported that NOD1 is widely distributed in various cell lines, mammalian tissues (40), and fish tissues (41, 42). In our study, rNOD1 was broadly expressed in all tested rabbit tissues. NOD1 is important for defense against bacterial invasion in intestinal and lung epithelial cells infected with *E. coli* (17, 29), it seem to be ubiquitously expressed in various tissues to modulate antibacterial activity. Moreover, iE-DAP can induce NOD1 gene expression in immune cells (43). To examine whether infection with *E. coli* activates rNOD1 signaling, rabbits were infected with *E. coli* and expression of rNOD1 was evaluated. In response to *E. coli* infection, expression of rNOD1 increased in the liver, spleen, and kidney, suggesting that rNOD1 signaling participates in the defense against *E. coli* infection.

Nucleotide-binding oligomerization domain 1 is a member of the NLR family, whose members initiate signal transduction mechanisms that include the stimulation of NF-κB, stress kinases, IFN regulatory factors, and autophagy (44). In the current study, rNOD1 overexpression and knockdown demonstrated that full length rNOD1, but not rNOD1-CARD or rNOD1-delCARD can activate NF-κB, indicating that the CARD domain is necessary, but not sufficient to activate downstream NF-κB signaling pathways. rNOD1 is also able to activate the NF-κB signaling pathway in RK-13 cells infected with *E. coli*. The overexpression of NOD2 effector domains has been shown to induce the expression of proinflammatory cytokines, antibacterial peptide cathelicidin-2, and type I- and II-IFNs (45). The current study showed that overexpression of full length rNOD1 significantly increased the expression of proinflammatory cytokines (*Il1b, Il6, Il8, Ifn-γ*, and *Tnf*) and defensins (*Defb124, Defb 125, Defb128, Defb135*, and *Np5*) compared to overexpression of the effector domain of rNOD1. These results indicate that the full length of rNOD1 may play an essential role in inducing the production of cytokines and defensins, which display diverse antimicrobial activities against various microorganisms, including Gram-positive and Gram-negative bacteria, fungi, and viruses. By contrast, previous studies have suggested that, because the C-terminal regulatory domain of *Muscovy duck* (Md) MDA5 and MdRIG-I can self-repress, overexpression of the CARD of MdMDA5 and MdRIG-I can activate signaling pathways more strongly than overexpression of the full length protein (46, 47). The activation of NOD1 results in oligomerization mediated by the nucleotide-binding NACHT domain to create a platform for the activation of downstream signaling molecules (44). In mammals, the CARD of NOD1 is then able to bind the CARD of receptor-interacting protein 2 through a homophilic CARD-CARD interaction to induce NF-κB activation. The rNOD1-CARD and rNOD1-delCARD domains were thus necessary for that activation. All of the above findings may explain why the full length of rNOD1 shows the strongest activation ability.

Nucleotide-binding oligomerization domain 1 has been shown to play an important role in inhibiting *E. tarda* and inducing the expression of inflammatory cytokines (48). In the current study, overexpression of rNOD1 inhibited the growth of *E. coli*, whereas knockdown of rNOD1 promoted the growth of *E. coli*. In addition, stimulation with iE-DAP inhibited the growth of *E. coli*. Overexpression of rNOD1 also increased the expression of *E. coli*-induced proinflammatory cytokines and β-defensins, whereas the knockdown of rNOD1 impaired these mRNAs. Importantly, in the context of NF-κB signaling inhibition, rNOD1 was unable to inhibit the growth of *E. coli* and downregulated the expression of proinflammatory cytokines and defensins. Such results suggest that the antibacterial activity and induction of immune-related genes for rNOD1 is mediated through NF-κB signaling. Autophagy is an important aspect of the innate immune response. Early resistance to invasive bacteria requires two processes, phagocytosis of bacteria followed by autophagy. The role of autophagy in protecting mammalian cells from multiple bacterial infections has been previously demonstrated. After invading an epithelial cell, group A *Streptococcus* is engulfed by autophagosomes, which fuse with the lysosomes to eliminate the bacteria (49). *Salmonella enterica* serovar Typhimurium escaping from vacuoles to the cytoplasm can be similarly eliminated by autophagosomes (50). Upregulation of autophagy promotes the killing of *Mycobacterium tuberculosis* (51), whereas inhibition of autophagy increases enterotoxigenic *E. coli*-induced cell death (52). The current study demonstrated that rNOD1 colocalized with the autophagy marker LC3, upregulated autophagy pathway protein LC3-II, and increased autolysosome formation in RK-13 cells infected with *E. coli*. Our observations concur with recent findings that NOD triggering activates autophagy, which results in the engulfment of intracellular bacteria by autophagosomes (53). These results indicate that rNOD1 not only plays an important role in inhibiting *E. coli*, but also induces proinflammatory cytokines and defensins, which are, in turn, involved in initiating inflammation and the innate host defense response (54).

Recently, it has been reported that coinjection of NOD ligands encapsulated with antigen significantly increases antibody response compared with the general adjuvant (55). In the current study, stimulation of rNOD1 with iE-DAP significantly induced the expression of proinflammatory cytokines and defensins in *E. coli*-infected RK-13 cells. The results indicated that new *E. coli* vaccine formulations may benefit from incorporation of an rNOD1 agonist (e.g., iE-DAP) to exploit the synergistic effects on cytokine and defensin production and to generate effective immune responses. Additionally, rNOD1 showed excellent antibacterial ability in the current study and eukaryotic or prokaryotic expression of rNOD1 could represent a novel therapeutic approach to target bacteria.

In conclusion, the rNOD1 gene was cloned from RK-13 cells. The protein contained evolutionarily conserved domains and was widely expressed in the tissues of rabbits. rNOD1 activated NF-κB signaling to induce the production of proinflammatory cytokine (*Il1b, Il6, Il8, Ifn-γ*, and *Tnf*) and defensins (*Defb124, Defb125*, and *Defb128*) in *E. coli-*infected cells. In addition, rNOD1 induced autophagy and played an important role in the inhibition of *E. coli*. rNOD1 may represent as new targets for vaccine adjuvant and drug development.

## Ethics Statement

This study was carried out in accordance with the recommendations of Shandong Agricultural University Animal Care and Use Committee (no. SDAUA-2015-005). The rabbits were purchased from the company, and the owners agreed that they can be used in our research.

## Author Contributions

MG and FW designed the experiments and wrote the article. ZZ and GH carried out most of the experiments. RL, NL, and YS collected and analyzed data. TC and LW developed the idea for the study and revised the article.

## Conflict of Interest Statement

The authors declare that the research was conducted in the absence of any commercial or financial relationships that could be construed as a potential conflict of interest.
